# Analysis of the safety and efficacy of PD-1/PD-L1 inhibitors combined with chemotherapy in the treatment of locally advanced resectable esophageal squamous cell carcinoma: a systematic review and meta-analysis based on four randomized controlled trials

**DOI:** 10.3389/fonc.2025.1590111

**Published:** 2025-08-13

**Authors:** Tao Luo, Hao Yang, Xingqiang Ran, Binwen Xu, Yue Zhang, Liwen Zhang, Chengcheng Zhang, Maoyong Fu

**Affiliations:** Department of Thoracic Surgery, North Sichuan Medical College Affiliated Hospital, Nanchong, Sichuan, China

**Keywords:** esophageal cancer, neoadjuvant chemoimmunotherapy, neoadjuvant chemotherapy, randomized controlled trials, meta-analysis

## Abstract

**Background:**

Esophageal cancer is a leading type of cancer globally. Most patients diagnosed with esophageal cancer present at a locally advanced stage, for which the standard treatment paradigm involves a multimodal approach combining neoadjuvant therapy with surgical resection. However, even under this regimen, 30%–40% of patients develop distant metastases postoperatively. This underscores an urgent need to refine existing therapeutic strategies and develop innovative multimodal protocols to address persistent oncological challenges in this high-risk population. In recent years, the advent of immunotherapy has expanded its application from advanced to early-stage settings, with neoadjuvant immunotherapy gaining traction in the management of locally advanced esophageal cancer. However, critical uncertainties persist regarding its preoperative use. This study aims to conduct a meta-analysis comparing the efficacy and safety of neoadjuvant chemoimmunotherapy (nICT) versus conventional neoadjuvant chemotherapy (nCT) in this patient population.

**Methods:**

A comprehensive literature retrieval strategy was implemented across PubMed (NLM), Embase (Elsevier), and the Cochrane Central Register of Controlled Trials, targeting studies published prior to December 2024 that compared novel immunochemotherapy (nICT) with conventional nCT in locoregionally advanced esophageal carcinoma. Pooled statistical analysis of the eligible randomized controlled trials (RCTs) was subsequently conducted to evaluate comparative safety and efficacy profiles.

**Results:**

The final analysis incorporated four randomized controlled trials (RCTs) comprising a total cohort of 629 participants. Patients receiving nICT for locoregionally advanced esophageal carcinoma demonstrated significantly prolonged intervals between final preoperative treatment and definitive surgical resection when compared to those undergoing conventional nCT alone [weighted mean difference (WMD): 0.71 weeks; 95% confidence interval (CI) 0.39–1.02; *P* < 0.0001]. The combined treatment showed significant advantages in pathological complete response (PCR) [odds ratio (OR): 3.44; 95% CI 1.98–5.97; *P* < 0.0001] and major pathological response (MPR) (OR: 2.56; 95% CI 1.23–5.30; *P* = 0.01). However, the incidence of anemia as an adverse reaction was higher in the combined treatment group (OR: 1.83; 95% CI 1.08–3.09; *P* = 0.02).

**Conclusion:**

Neoadjuvant chemotherapy combined with immunotherapy for treating locally advanced esophageal cancer is effective and safe. However, due to the absence of long-term follow-up data, additional large-scale, multicenter randomized controlled trials are required to confirm these results.

## Introduction

Esophageal cancer is a digestive tract tumor with a high incidence, malignancy, and mortality rate. It is one of the seven most common cancers and the sixth leading cause of cancer-related deaths globally (1). Esophageal cancer can be classified into two main histological types: esophageal squamous cell carcinoma (ESCC) and esophageal adenocarcinoma. In Asian populations, ESCC accounts for approximately 90% of esophageal cancer cases. The majority of esophageal cancer patients are diagnosed at the locally advanced stage, with a 5-year survival rate of only 20% (2, 3). As a result, esophageal cancer poses a significant threat to human health.

Surgery remains the primary treatment for esophageal cancer, but surgery alone often does not yield satisfactory results. According to the results of the CROSS (4, 5), NEOCRTEC5010 (6, 7), and JCOG1109 (8) trials, neoadjuvant therapy [such as neoadjuvant chemotherapy (nCT) or neoadjuvant chemoradiotherapy (nCRT)] combined with surgery is the standard treatment for locally advanced ESCC. However, patients who undergo nCT or nCRT combined with esophagectomy still have unsatisfactory long-term survival rates; thus, there is a need for a new, more effective, and less toxic neoadjuvant treatment plan to enhance clinical outcomes for patients with locally advanced esophageal cancer.

With the development of immunotherapy, immune checkpoint inhibitors have changed the treatment landscape for esophageal cancer patients. The most common of these are programmed cell death receptor-1 (PD-1) and programmed cell death ligand-1 (PD-L1). According to studies such as ESCORT (9) and KEYNOTE-590 (10), immunotherapy combined with chemotherapy has provided significant survival benefits for patients with advanced esophageal cancer. As a result, many researchers have started combining immunotherapy with chemotherapy as a preoperative neoadjuvant treatment for locally advanced esophageal cancer. With the completion of phase II clinical trials, many studies have reported the safety and efficacy of neoadjuvant chemotherapy combined with immunotherapy. Although network meta-analyses (11–13) have been conducted on this topic, the studies included in those analyses were mostly non-randomized controlled trials and did not include recently published research. Compared to non-randomized trials, randomized controlled trials (RCTs) offer superior control of confounding factors. Through randomization, RCTs prevent researchers or participants from selectively assigning groups based on preference or judgment, thereby minimizing selection bias. By controlling confounders, establishing robust comparators, and reducing systematic biases, RCTs provide the highest level of validity for causal inference. Thus, this study intends to offer dependable evidence for preoperative neoadjuvant treatment protocols for patients with locally advanced esophageal cancer by incorporating four randomized controlled trials to compare the effectiveness of chemotherapy combined with immunotherapy versus neoadjuvant chemotherapy alone (14–17).

## Methods

Prior to literature retrieval, the review protocol was registered on PROSPERO (CRD42025647824), ensuring alignment with PRISMA 2020 standards for transparent meta-analytic reporting.

### Search strategy

Following PRISMA-Search guidelines, systematic searches were performed using Boolean logic operators (AND/OR/NOT) in the following platforms: 1) PubMed, 2) Embase, and 3)Cochrane Library. Medical subject headings (MeSH) were used to search for the following terms: esophageal cancer, neoadjuvant, preoperative, programmed cell death-1 (PD-1), programmed cell death ligand-1 (PD-L1), and immunotherapy [including all known immune checkpoint inhibitors (ICIs): pembrolizumab, toripalimab, socazolimab, camrelizumab]. High-quality clinical trials published from 2020 to 2025 were selected, with no restrictions on publication year or country. In addition, reference lists from original articles and review articles were manually filtered to retrieve studies not identified in the database search.

### Inclusion and exclusion criteria

The studies were selected based on the following criteria: i) inclusion of resectable stage II–IVa ESCC confirmed by histopathological examination of tissue samples; ii) inclusion of RCTs comparing neoadjuvant immunotherapy combined with chemotherapy (nICT) and standard neoadjuvant chemotherapy (nCT) for the treatment of ESCC; iii) evaluation of the efficacy and safety of different neoadjuvant treatment regimens, with primary comparative endpoints; and iv) the included RCTs must report at least one clinical outcome measure, including major pathological response (MPR), treatment-related adverse events (TRAEs), pathological complete response (PCR), serious adverse events (SAEs), R0 resection rate, and postoperative complication rates. The exclusion criteria were as follows: i) non-randomized controlled trials; ii) RCTs with unclear clinical outcome measures; iii) RCTs where the experimental group’s neoadjuvant therapy is not chemotherapy combined with immunotherapy and the control group’s neoadjuvant therapy is not standard neoadjuvant chemotherapy; and iv) studies including fewer than 10 patients for analysis.

### Data screening

The data screening was independently performed by two authors, who extracted relevant data from the studies that met the inclusion criteria. When studies had overlapping cohorts, the one with the largest sample size or the most comprehensive data was chosen for analysis. In the event of discrepancies during the data selection process, the two authors would resolve them through discussion. If a resolution could not be reached, the final decision would be made by the corresponding author. The extracted data included the following: 1) data extraction: first author, publication year, study design, sample size, and neoadjuvant treatment regimen; 2) baseline characteristics of the enrolled patients: gender, age, and clinical TNM stage; 3) outcome data: MPR, PCR, R0 resection rate, postoperative complications (anastomotic leakage, pneumonia), and the incidence of grade 3–4 adverse events.

### Assessment of the quality of included studies

Two independent reviewers assessed the risk of bias in the included RCTs using the Cochrane Risk of Bias Tool (18). This tool includes six items: random sequence generation, allocation concealment, blinding, completeness of outcome data, selective reporting of outcomes, and other sources of bias. Based on these six items, the risk of bias was classified into three levels: “high risk,” “unclear risk,” and “low risk.”

### Statistical analysis

The meta-analysis was performed using Review Manager 5 software (RevMan 5.3, Cochrane Community, London, UK). Statistical heterogeneity was assessed using Higgins *I*², which indicates the percentage of total variation across studies. When *I*² is less than 50%, a fixed-effect model (Mantel–Haenszel method) was used to combine homogeneous studies. Otherwise, a random-effects model (DerSimonian–Laird) was applied. The effect measures for quantitative data were mean difference (MD) with 95% confidence intervals (CI), and for qualitative data, odds ratio (OR) with 95% CI. A *P*-value of <0.05 was considered statistically significant. The median and interquartile range for continuous variables were converted to mean values and standard deviations using the sample mean estimation method (19) and standard deviation estimation method (20), respectively, via an online tool (http://www.comp.hkbu.edu.hk/~xwan/median2mean.html).

### Sensitivity analysis

We employed the leave-one-out method to remove studies individually from the pooled effect in order to assess the stability of the estimates. Furthermore, we evaluated the robustness by considering study cohort size (excluding studies with fewer than 100 patients), as this could impact heterogeneity. However, sensitivity analysis could not be performed for three or fewer studies.

### Publication bias

When fewer than 10 studies were included, the test power was inadequate. As a result, we did not conduct any further publication bias analysis (21, 22).

## Results

### Basic characteristics of the included studies

**Figure 1:**
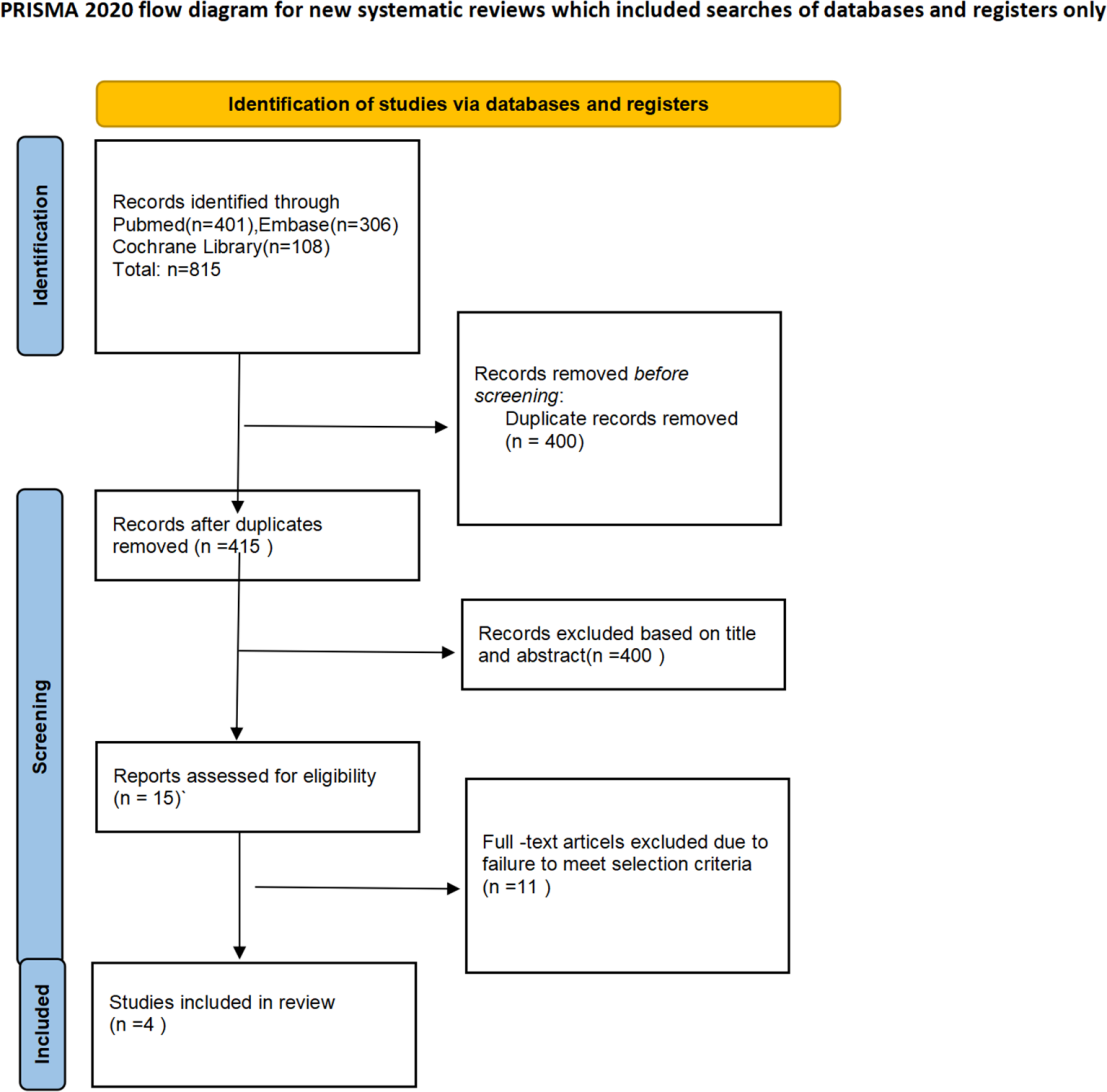
PRISMA flowchart.

The PRISMA flow diagram of the study selection process is shown in **Figure 1**. Based on the search strategy, a total of 815 articles were identified. After removing 400 duplicates, 415 studies remained for screening. Of these, 400 articles (including conference abstracts, letters, case reports, or related studies) were excluded from the screening. The full texts of the remaining 15 studies were further screened, with 11 studies excluded due to lack of data specificity, non-adult participants, incorrect interventions, and other factors. Finally, four studies were included in the final meta-analysis. All the included studies were conducted in China. Among the four studies, all were randomized controlled trials, including two phase III trials and one phase II trial. All studies used PD-1 or PD-L1 inhibitors. The most common nCT regimen was paclitaxel or nab-paclitaxel combined with carboplatin or cisplatin. The main characteristics of the included studies are shown in **Table 1**, and the primary outcomes are presented in **Table 2**.

**Table 1:** Basic characteristics of the included literature.

First author (year) / Country	Study type	Study center	Sample size	Clinical staging	Intervention	ICI drugs	CT regimen	Neoadjuvant cycle	Histological type
Yan Zheng (2024) (17)/china	RCT	Single	252	cT1N1-3M0 OR T2-3N0-3M0	NICT	toripalimab	TP	2	ESCC
Yong Li (2023) (14)/china	RCT	Multicenter	64	cT2NxM0 OR T3-4aNxM0	NICT	Socazolimab	TP	4	ESCC
Jianjun Qin (2024) (16)/china	RCT	Multicenter	259	cT1b-3N1-3M0 OR T3N0M0	NICT	camrelizumab	TP	2	ESCC
Bingjiang Huang (2021) (15)/china	RCT	Single	54	II-IVA期	NICT	pembrolizumab	docetaxel+nidaplatin	2	ESCC

RCT, randomized controlled trial; nICT, neoadjuvant immune checkpoint inhibitors combined with chemotherapy; ICI, immune checkpoint inhibitor; CT, chemotherapy; TP, paclitaxel + platinum-based drugs; ESCC, esophageal squamous cell carcinoma.

**Table 2:** Main data extracted from the studies.

	Time from last neoadjuvant dose to definitive surgery(weeks)		Duration of surgery(min)		Number of resected lymph nodes		Incidence of Postoperative complication	
Author (year)	Combined group	Chemotherapy group	Combined group	Chemotherapy group	Combined group	Chemotherapy group	Combined group	Chemotherapy group
Yan Zheng (2024) (17)/china	4.4944±1.2784	3.4012±2.638	303.5191±52.6392	300.5283±66.7031	25.4±7.92	24.7±10.06	91/102	78/88
Jianjun Qin (2024) (16)/china	6.0515±1.7992	5.5406±1.0496	256.5177±50.9648	267.0435±91.5988	37.35±15.74	34.81±13.5	45/116	33/103
Yong Li (2023) (14)/china	7.7672±3.4891	6.5682±1.2683	269.4603±99.4428	240.5475±60.4553	42.91±14.56	40.46±20.48	2/29	2/29
Bingjiang Huang (2021) (15)/china	NR	NR	NR	NR	NR	NR	NR	NR

	R0 resection rate		PCR rate		MPR rate		Incidence of Pneumonia	
Author (year)	Combined group	Chemotherapy group	Combined group	Chemotherapy group	Combined group	Chemotherapy group	Combined group	Chemotherapy group
Yan Zheng (2024) (17)/china	102/102	88/88	19/102	4/88	30/102	6/88	78/102	58/88
Jianjun Qin (2024) (16)/china	111/116	95/103	20/130	6/129	47/130	27/129	21/116	15/103
Yong Li (2023) (14)/china	29/29	28/29	12/29	8/29	20/29	18/29	1/29	1/29
Bingjiang Huang (2021) (15)/china	21/21	26/27	7/23	3/31	NR	NR	NR	NR

	Incidence of TRAE		Incidence of Grade 3 or 4 TRAE		Incidence of Decreased neutrophil count		Incidence of Anastomotic fistula	
Author (year)	Combined group	Chemotherapy group	Combined group	Chemotherapy group	Combined group	Chemotherapy group	Combined group	Chemotherapy group
Yan Zheng (2024) (17)/china	120/120	129/129	12/120	16/129	NR	NR	6/102	6/88
Jianjun Qin (2024) (16)/china	108/130	104/125	38/120	36/125	48/130	38/125	5/116	6/103
Yong Li (2023) (14)/china	32/32	32/32	21/32	20/32	25/32	25/32	1/29	0/29
Bingjiang Huang (2021) (15)/china	NR	NR	NR	NR	19/23	19/31	NR	NR

	Incidence of Decreased white blood cell count		Incidence of Anaemia		Incidence of Decreased platelet count		Incidence of Nausea	
Author (year)	Combined group	Chemotherapy group	Combined group	Chemotherapy group	Combined group	Chemotherapy group	Combined group	Chemotherapy group
Yan Zheng (2024) (17)/china	NR	NR	NR	NR	NR	NR	101/120	108/129
Jianjun Qin (2024) (16)/china	51/130	41/125	30/130	23/125	8/130	5/125	28/130	33/125
Yong Li (2023) (14)/china	25/32	22/32	32/32	27/32	32/32	27/32	13/32	10/32
Bingjiang Huang (2021) (15)/china	19/23	19/31	19/23	18/31	13/23	12/31	12/23	12/31

	Incidence of Vomiting		Incidence of Fatigue		Incidence of Hair loss			
Author (year)	Combined group	Chemotherapy group	Combined group	Chemotherapy group	Combined group	Chemotherapy group		
Yan Zheng (2024) (17)/china	42/120	39/129	115/120	116/129	107/120	114/129		
Jianjun Qin (2024) (16)/china	9/130	18/125	10/130	11/125	30/130	25/125		
Yong Li (2023) (14)/china	6/32	7/32	12/32	8/32	10/32	9/32		
Bingjiang Huang (2021) (15)/china	8/23	10/31	20/23	22/31	NR	NR		

1) Data not reported are indicated as NR. 2) Continuous variables are presented as mean ± standard deviation. 3) Binary variables are presented as the number of patients with complications/the number of patients without complications.

### Sensitivity analysis and quality assessment

**Figure 2:**
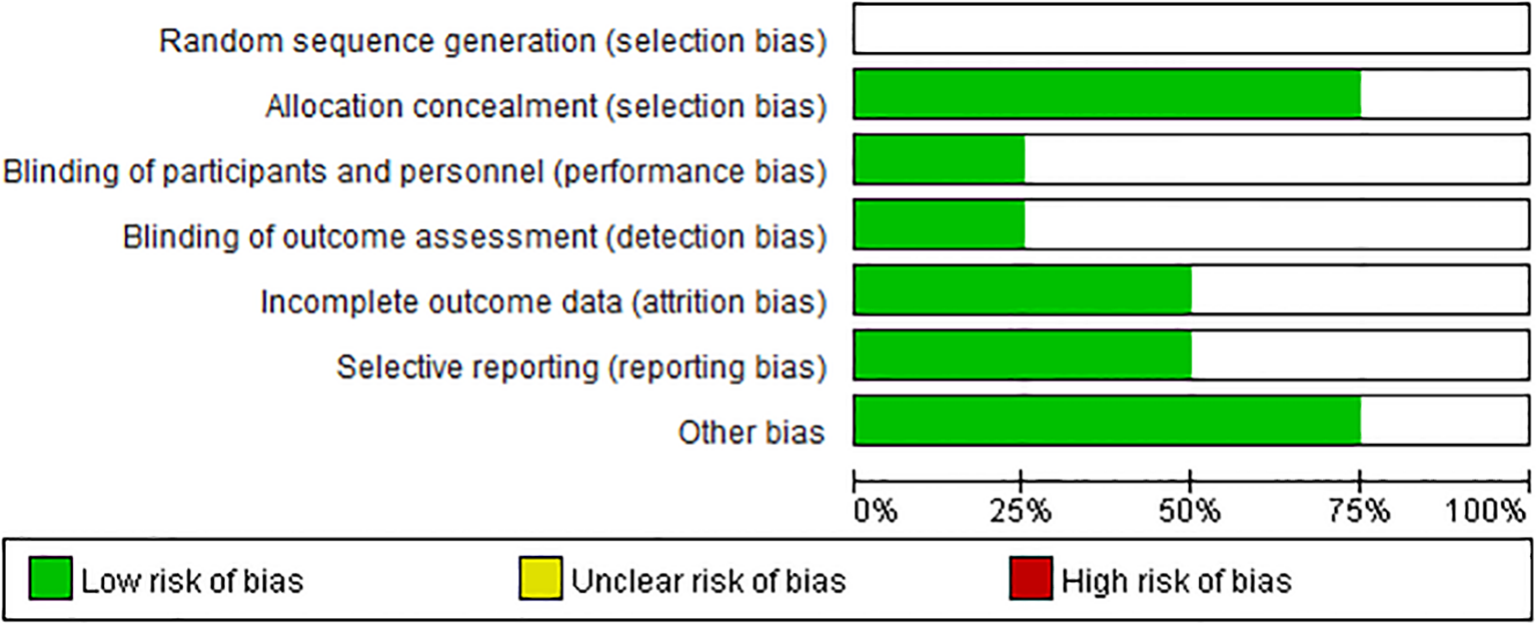
Risk of bias graph: a review of the authors’ judgments on each risk of bias item, expressed as a percentage in all the included studies.

**Figure 3:**
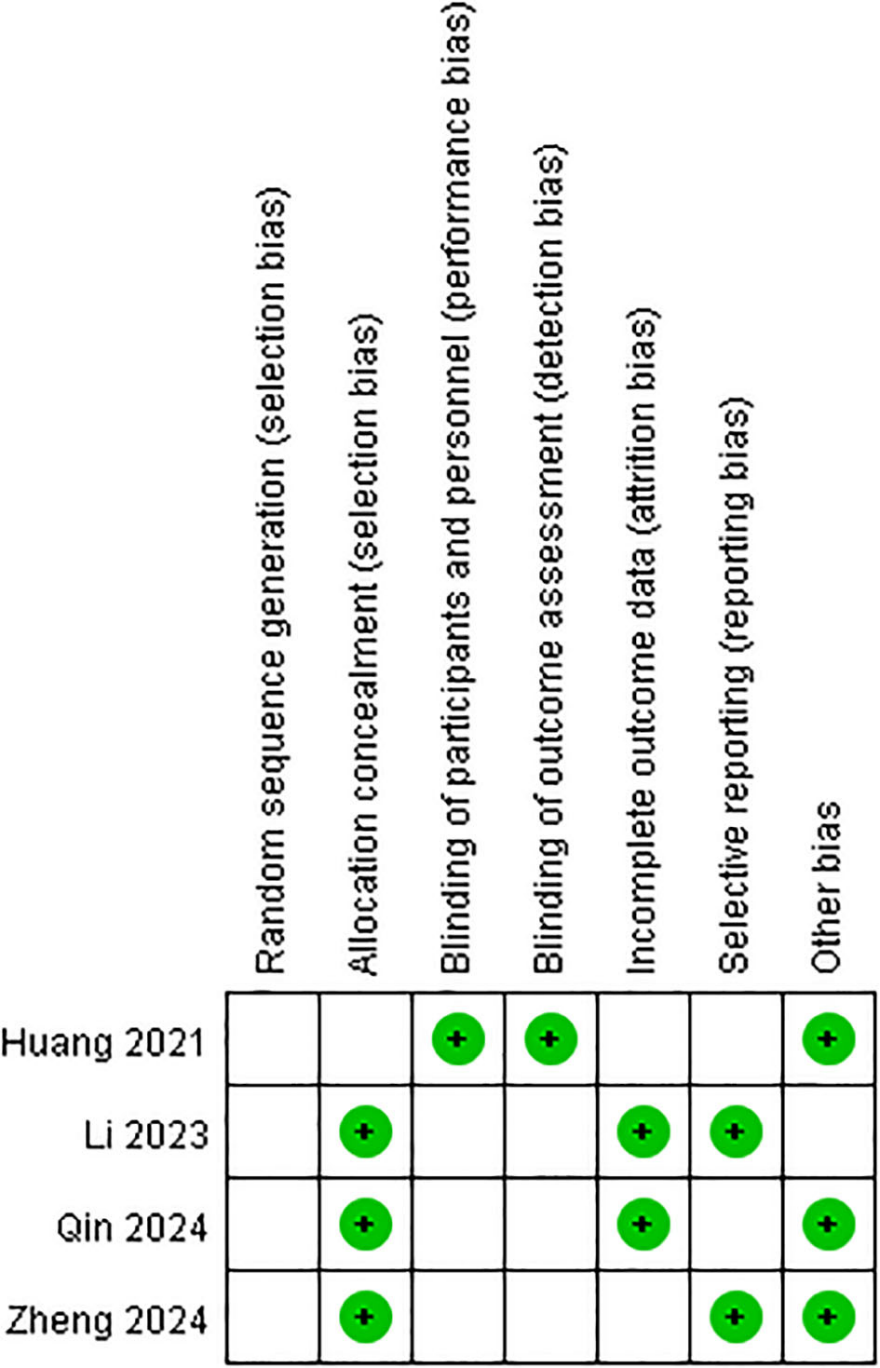
Risk of bias summary: the authors’ judgment on each methodological quality item for each included study is reviewed. The symbols “+” (low risk of bias), “−” (high risk of bias), and “blank” (uncertain risk of bias) are used to represent these judgments.

In this meta-analysis, we performed a leave-one-out analysis by sequentially excluding individual studies for each extracted outcome measure before conducting the combined analysis. The results showed little variation, indicating stability. Additionally, we summarized the risk of bias for the four studies, as shown in **Figures 2**, **3**. Most of the included RCTs were assessed as having a low risk of bias, suggesting that the randomized controlled trials included in the study were of high quality.

### Primary outcomes

#### Preoperative neoadjuvant adverse reactions

**Figure 4:**
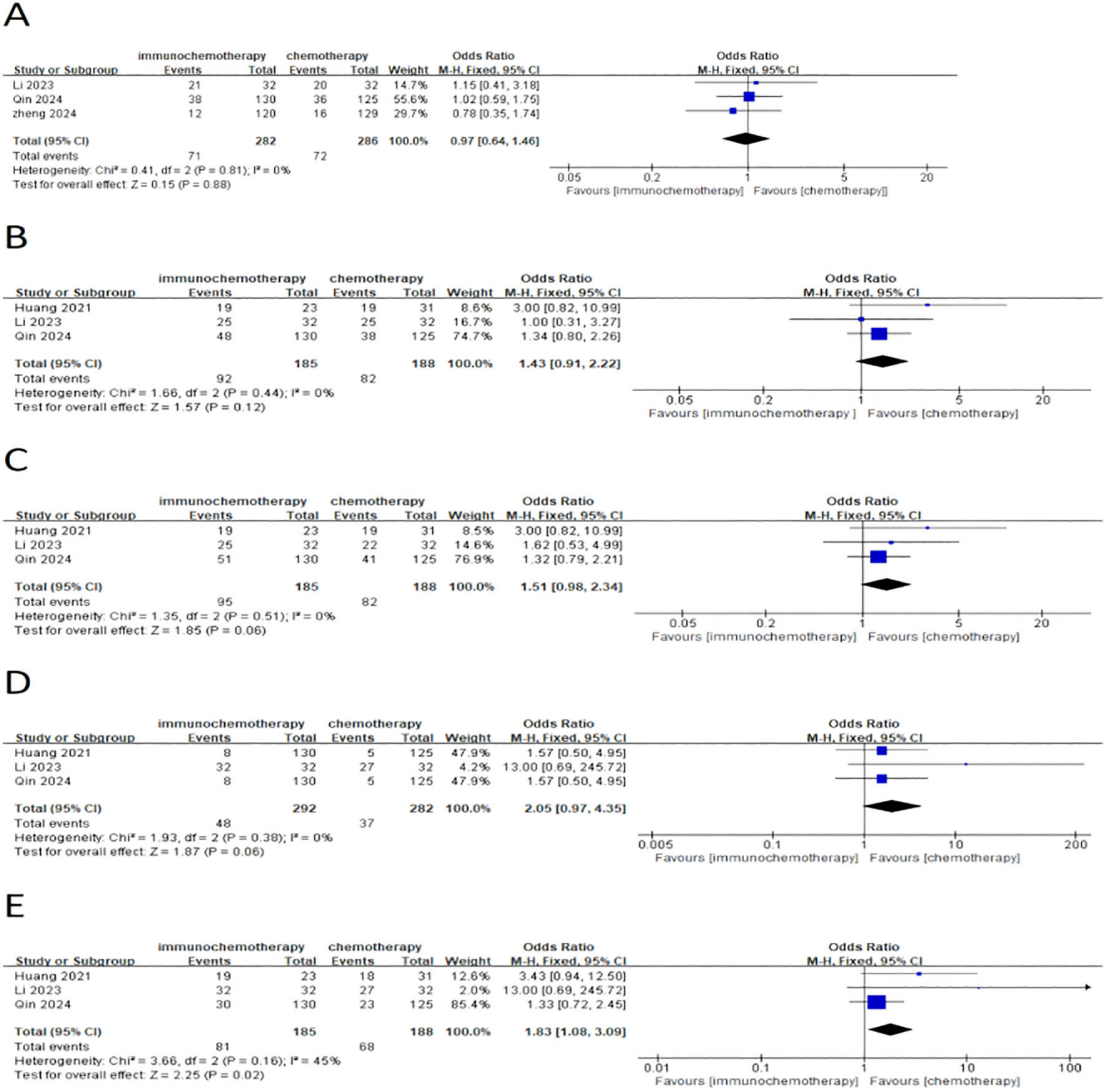
Forest plot comparing neoadjuvant chemotherapy combined with immunotherapy (neoadjuvant chemo-immunotherapy group) and neoadjuvant chemotherapy alone (neoadjuvant chemotherapy group) in esophageal squamous cell carcinoma: **(A)** grade 3–4 adverse reactions; **(B)** neutropenia; **(C)** leukopenia; **(D)** thrombocytopenia; and **(E)** anemia.

**Figure 5:**
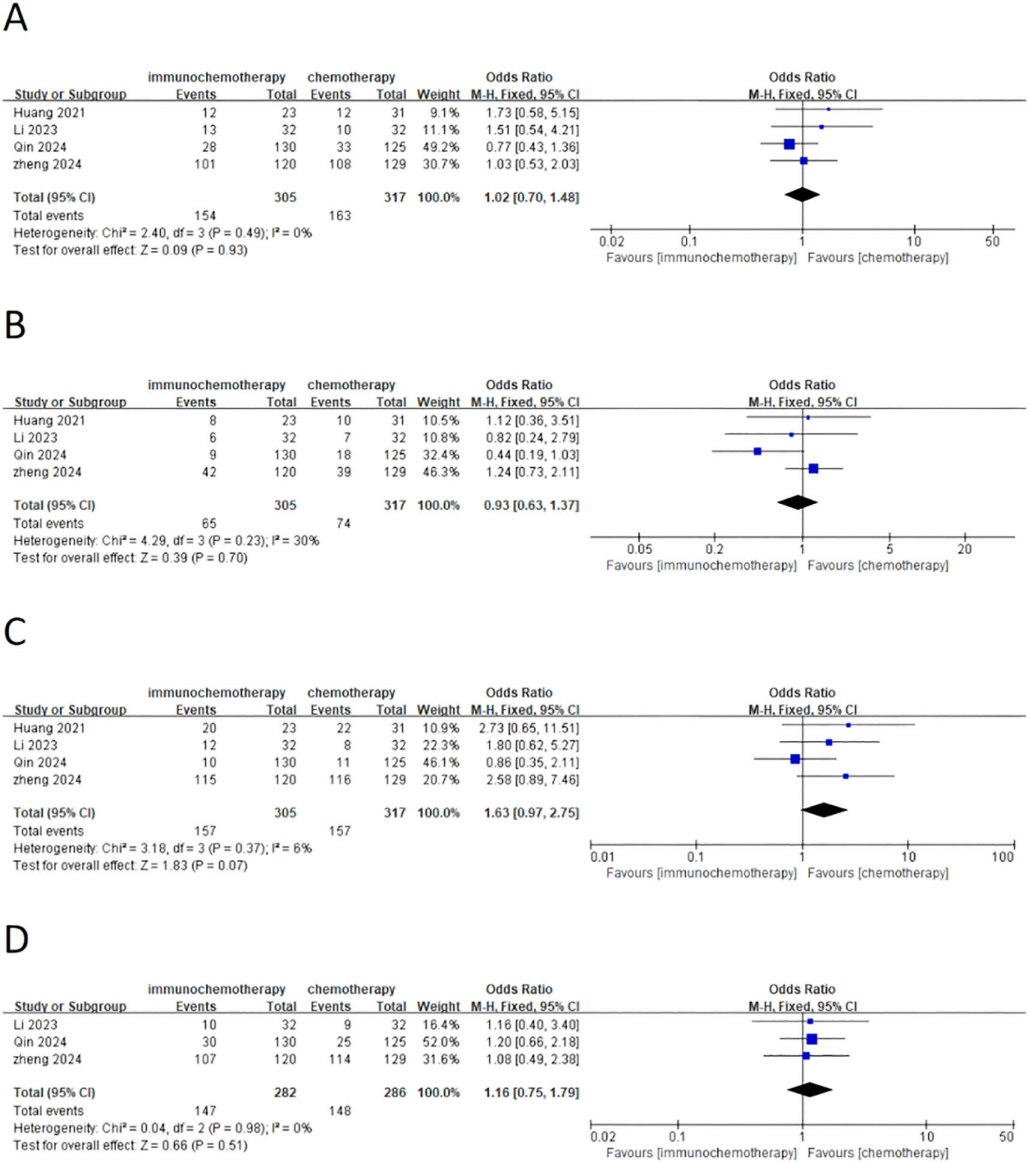
Forest plot comparing neoadjuvant chemotherapy combined with immunotherapy (neoadjuvant chemo-immunotherapy group) and neoadjuvant chemotherapy alone (neoadjuvant chemotherapy group) in esophageal squamous cell carcinoma: **(A)** nausea; **(B)** vomiting; **(C)** fatigue; **(D)** hair loss.

This meta-analysis included a total of four studies, three of which reported the incidence of grade 3–4 adverse reactions in a total of 586 patients. Among them, 282 patients were in the neoadjuvant chemotherapy combined with immunotherapy group, and 286 patients were in the neoadjuvant chemotherapy group. The heterogeneity between studies was very low (*I*² = 0), so a fixed-effects model was used for analysis. The results showed that neoadjuvant chemotherapy combined with immunotherapy did not significantly increase the incidence of grade 3 adverse reactions compared to neoadjuvant chemotherapy alone (OR = 0.97; 95% CI 0.64–1.46; *P* = 0.88) (**Figure 4A**). Additionally, three studies involving 373 patients reported adverse reactions such as neutropenia, leukopenia, thrombocytopenia, and anemia. Since the heterogeneity of the included studies was very low, a fixed-effects model was applied. The results indicated a significant difference in the incidence of adverse reactions, except for anemia (**Figure 4E**) (OR = 1.83; 95% CI 1.08–3.09; *P* = 0.02). For neutropenia (**Figure 4B**) (OR = 1.43; 95% CI 0.91–2.22; *P* = 0.12), leukopenia (**Figure 4C**) (OR = 1.51; 95% CI 0.98–2.34; *P* = 0.06), and thrombocytopenia (**Figure 4D**) (OR = 2.05; 95% CI 0.97–4.35; *P* = 0.06), no significant differences were found in the incidence of adverse events. For adverse reactions like nausea, vomiting, and fatigue caused by neoadjuvant treatment, this meta-analysis included four studies with a total of 622 patients. Since the heterogeneity between studies was low, a fixed-effects model was used. The results showed no significant differences in adverse events [nausea: OR = 1.02; 95% CI 0.70–1.48; *P* = 0.93 (**Figure 5A**), vomiting: OR = 0.93; 95% CI 0.63–1.37; *P* = 0.70 (**Figure 5B**), and fatigue: OR = 1.63; 95% CI 0.97–2.75; *P* = 0.07 (**Figure 5C**)]. For hair loss, based on three studies, the meta-analysis showed no significant difference in the incidence of hair loss between neoadjuvant chemotherapy combined with immunotherapy and neoadjuvant chemotherapy (OR = 1.16; 95% CI 0.75–1.79; *P* = 0.51) (**Figure 5D**). This meta-analysis also analyzed immune-related adverse events (such as rash, abnormal thyroid function, elevated transaminase, diarrhea, elevated serum creatinine, and pneumonia) (**Table 3**). Overall, in the nICT group, grade 1–2 adverse events were relatively common, and the incidence of grade 3–4 adverse events was 1.63%. Among them, rash and thyroid dysfunction were the most common immune-related adverse events, with incidence rates of 11.15% and 15.41%, respectively. There were no reports of treatment-related deaths, nor were there any reports of delayed or non-operable surgeries due to severe immune-related adverse events.

**Table 3:** Immune-related adverse events.

	Rash		Abnormal thyroid function		Elevated transaminase		Diarrhea		Elevated serum creatinine		Pneumonia	
First author (year) / Country	all	≥ grade 3	all	≥ grade 3	all	≥ grade 3	all	≥ grade 3	all	≥ grade 3	all	≥ grade 3
Yan Zheng (2024) (17)/china	19	0	37	1	1	0	5	0	NR	NR	4	0
Yong Li (2023) (14)/china	1	0	2	0	0	0	NR	NR	1	0	NR	NR
Jianjun Qin (2024) (16)/china	9	1	4	0	3	2	2	1	2	0	NR	NR
Bingjiang Huang (2021) (15)/china	5	0	4	0	12	0	3	0	NR	NR	1	0

1) Data not reported are indicated as NR.

### Surgical-related data

**Figure 6:**
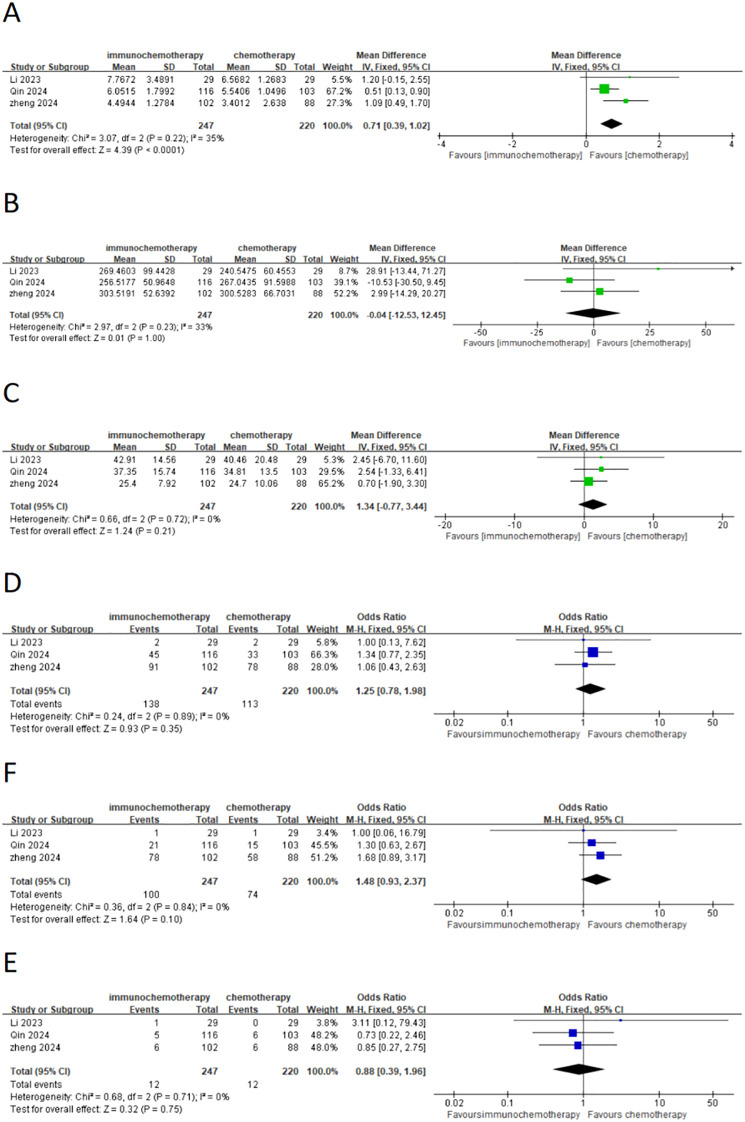
Forest plot comparing neoadjuvant chemotherapy combined with immunotherapy (neoadjuvant chemo-immunotherapy group) and neoadjuvant chemotherapy alone (neoadjuvant chemotherapy group) in esophageal squamous cell carcinoma: **(A)** time from last neoadjuvant dose to definitive surgery (weeks); **(B)** duration of surgery; **(C)** number of resected lymph nodes; **(D)** postoperative complication; **(E)** pneumonia; and **(F)** anastomotic fistula.

**Figure 7:**
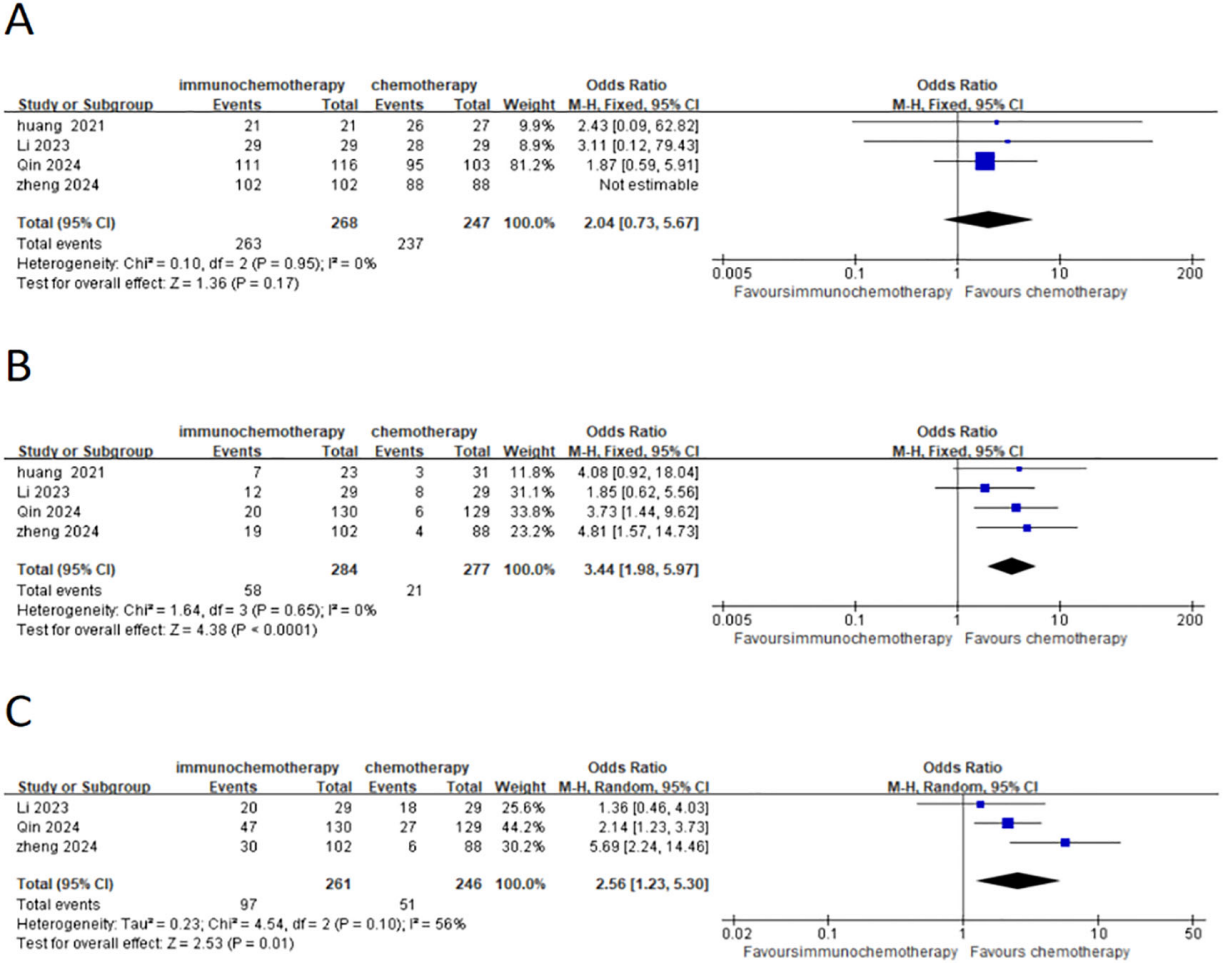
Forest plot comparing neoadjuvant chemotherapy combined with immunotherapy (neoadjuvant chemo-immunotherapy group) and neoadjuvant chemotherapy alone (neoadjuvant chemotherapy group) in esophageal squamous cell carcinoma: **(A)** R0 resection rate; **(B)** PCR (pathological complete response); and **(C)** MPR (major pathological response).

This meta-analysis included three studies that analyzed the interval from the last neoadjuvant treatment to surgery, the duration of surgery, the number of lymph nodes removed, and the overall incidence of postoperative complications. Additionally, it also analyzed common postoperative complications such as pneumonia and anastomotic leakage. A total of 467 patients were included, with 247 patients in the neoadjuvant chemotherapy combined with immunotherapy group and 220 patients in the neoadjuvant chemotherapy group. The heterogeneity between studies was very low (*I*² < 50%), so a fixed-effects model was used. The results showed that the interval from the last neoadjuvant treatment to surgery was shorter in the neoadjuvant chemotherapy combined with immunotherapy group compared to the neoadjuvant chemotherapy group (WMD = 0.71 weeks; 95% CI 0.39–1.02; *P* < 0.001) (**Figure 6A**). There was no significant difference between the groups in terms of the duration of surgery (**Figure 6B**), the number of lymph nodes removed (**Figure 6C**), or the incidence of postoperative complications (**Figure 6D**) including the incidence of postoperative pneumonia (**Figure 6E**) and anastomotic leakage (**Figure 6F**). MPR, defined as the presence of ≤10% residual tumor cells on postoperative pathological examination, was analyzed in three studies with a total of 507 patients, including 261 patients in the neoadjuvant chemotherapy combined with immunotherapy group and 246 patients in the neoadjuvant chemotherapy group. Due to high heterogeneity (*I*² = 56% > 50%), a random-effects model was used. The results showed a statistically significant difference in the overall MPR (OR = 2.56; 95% CI 1.23–5.30; *P* = 0.001) (**Figure 7C**). For pathological complete response (PCR), defined as the absence of residual tumor cells after the resection of the tumor and regional lymph nodes, four studies with 561 patients were included. Of these, 284 patients were in the neoadjuvant chemotherapy combined with immunotherapy group, and 277 patients were in the neoadjuvant chemotherapy group. The results showed a statistically significant difference in the overall PCR (OR = 3.44; 95% CI 1.98–5.97; *P* < 0.0001) (**Figure 7B**). The meta-analysis found that the PCR rate was higher in the neoadjuvant chemotherapy combined with immunotherapy group. However, in terms of R0 resection rate (defined as the complete removal of the tumor with negative microscopic margins, indicating no tumor residue, which is another important indicator for evaluating the effectiveness of neoadjuvant treatment), four studies involving 515 patients reported an R0 resection rate. The results showed that both groups (neoadjuvant chemotherapy combined with immunotherapy and neoadjuvant chemotherapy) had high R0 resection rates, with no statistically significant difference (OR = 2.04; 95% CI 0.73–5.67; *P* = 0.17) (**Figure 7A**).

## Discussion

Esophageal cancer represents a major global health concern, marked by both high incidence and mortality rates. Many patients with esophageal cancer are already diagnosed at a locally advanced stage. In East Asia, based on the results of the CROSS, NEOCRTEC5010, and JCOG1109 trials, neoadjuvant chemotherapy or neoadjuvant chemoradiotherapy is the standard treatment for resectable locally advanced esophageal cancer. However, despite the application of these two neoadjuvant modalities (nICT and nCT), approximately 30%–40% of patients still experience postoperative recurrence. This underscores the urgent need for novel therapeutic strategies to address this unresolved clinical challenge. In recent years, with the development of immunotherapy, neoadjuvant immunotherapy has been shown to significantly improve the PCR rate in esophageal cancer patients, and its toxicity is controllable (12, 23, 24). Although previous meta-analyses have confirmed the safety and efficacy of nICT, most of the studies included were retrospective, with varying conditions and inconsistent results. Therefore, combining the latest high-quality RCTs for meta-analysis is crucial in addressing this issue. In this study, by limiting the analysis to RCTs, we have reduced the influence of some potential confounding factors in previous network meta-analyses that were not adjusted for, thus making our estimates more reliable. The inclusion of newer studies allows for a more updated and comprehensive analysis.

This meta-analysis included four randomized controlled trials with a total of 629 patients, comparing the safety and efficacy of neoadjuvant chemotherapy combined with immunotherapy and neoadjuvant chemotherapy alone. In terms of safety, we explored the incidence of grade 3–4 adverse events, neutropenia, leukopenia, anemia, thrombocytopenia, nausea, vomiting, fatigue, etc. The results showed that the incidence of grade 3–4 adverse reactions was low in both groups, but the difference was not statistically significant. Furthermore, we analyzed the changes in blood parameters such as neutrophils, leukocytes, red blood cells, and platelets. Consistent with previous meta-analyses, no significant differences were observed in neutrophils, leukocytes, and platelets. However, regarding anemia, our analysis yielded different results from previous meta-analyses, showing that the incidence of anemia in the neoadjuvant chemotherapy combined with immunotherapy group was higher than that in the chemotherapy group, and the difference was statistically significant. This may be due to the potential inhibitory effects of immune checkpoint inhibitors on B cells, NK cells, and macrophages, which could lead to the release of T lymphocytes/NK cell toxicity and B cells producing autoantibodies, resulting in reduced blood cells (25). However, given the limited number of studies, additional large-scale randomized controlled trials are necessary to validate these findings. Overall, the inclusion of immunotherapy with chemotherapy does not notably raise the incidence of chemotherapy-related side effects. However, close monitoring and management of blood system changes should not be neglected in clinical practice. Meanwhile, we conducted a comprehensive statistical analysis of the incidence of immune-related adverse events (irAEs). Grade 1–2 adverse events were the most prevalent, accounting for the majority of cases, while grade ≥3 adverse events occurred in less than 5% of patients. The most frequently observed immune-related adverse events were rash and abnormal thyroid function. Notably, there were no fatalities or delayed surgeries attributable to immune-related adverse events, further substantiating the safety of incorporating immune checkpoint inhibitors into chemotherapy regimens. Additionally, this meta-analysis analyzed postoperative complications, including pneumonia and anastomotic leakage, and showed no significant differences between the two groups. These results are consistent with previous meta-analyses, further indicating that adding immunotherapy to neoadjuvant chemotherapy does not increase postoperative complications, supporting the safety of the combination therapy. In terms of efficacy, this meta-analysis focused on R0 resection rate, PCR rate, and MPR rate. The R0 resection rate is a standard for evaluating the effect of surgical intervention in esophageal cancer and is closely related to patient prognosis. The results indicated no significant difference in the R0 resection rate between the two groups, indicating that neoadjuvant treatment, whether neoadjuvant chemotherapy combined with immunotherapy or chemotherapy alone, is effective for complete tumor resection with negative margins in esophageal cancer patients. The PCR and MPR rates reflect the tumor regression after neoadjuvant therapy and are important indicators of the efficacy of neoadjuvant treatment. In this meta-analysis, the PCR rate in the neoadjuvant chemotherapy combined with immunotherapy group was significantly higher than in the chemotherapy group (OR = 3.44; 95% CI 1.98–5.97; *P* < 0.0001), and the same result was observed for the MPR rate (OR = 2.56; 95% CI 1.23–5.30; *P* = 0.001). These results are consistent with previous meta-analyses, further confirming that combining immunotherapy with neoadjuvant chemotherapy increases the likelihood of achieving complete pathological response and significant tumor regression, validating the efficacy of combining immunotherapy with neoadjuvant chemotherapy. Furthermore, in our analysis of the interval from the last neoadjuvant treatment to surgery, we found that the interval in the neoadjuvant chemotherapy combined with immunotherapy group was longer (WMD = 0.71 weeks; 95% CI 0.39–1.02; *P* < 0.001), which may be due to the additional time required for immune therapy. Additionally, the interval is largely influenced by subjective patient preferences and other factors. In terms of surgery duration and lymph node dissection, our study showed no significant difference between the two groups, suggesting that adding immunotherapy to neoadjuvant chemotherapy does not increase the difficulty of the surgical procedure. Overall, the safety of combining neoadjuvant chemotherapy with immunotherapy is acceptable.

This meta-analysis has a few limitations. First, the studies included did not have a clear consensus on the choice of immunotherapy, and the specific immunosuppressive agents used varied depending on individual patients’ conditions. Second, the limited number of studies and patients included in the analysis resulted in high heterogeneity for some outcomes. Third, some of the included studies have not yet reached their primary endpoints, and complete data from these studies were unavailable, making it challenging to analyze survival outcomes (such as PFS and OS). However, we believe that the results presented here are sufficient until more long-term data and follow-up results from randomized controlled trials become available. These future studies can link neoadjuvant immunotherapy’s pathological response with OS and PFS and explore its clinical and safety outcomes. Fourth, this meta-analysis only focused on patients with ESCC, and there is still relatively little evidence regarding neoadjuvant immunotherapy in esophageal adenocarcinoma (EAC). More clinical trials are needed, especially in Europe and the United States, where the incidence of EAC is relatively high. Fifth, all the included studies were conducted in China, introducing some regional bias.

## Conclusion

This meta-analysis confirms the effectiveness and safety of combining neoadjuvant chemotherapy with immunotherapy for the treatment of locally advanced esophageal squamous cell carcinoma. Compared with neoadjuvant chemotherapy alone, the combination therapy resulted in higher PCR and MPR rates. However, due to the limited research on this combination therapy and the lack of long-term follow-up results, it is difficult to link the higher PCR and MPR rates with long-term benefits. Additionally, regarding anemia, we obtained different results from previous meta-analyses. These findings lay the foundation for future research, but long-term follow-up, large-scale, and multicenter randomized controlled trials are needed to validate and refine these results.

## Data Availability

The original contributions presented in the study are included in the article/**Supplementary Material**. Further inquiries can be directed to the corresponding author.
