# Supplementary material for: Analysis of the safety and efficacy of PD-1/PD-L1 inhibitors combined with chemotherapy in the treatment of locally advanced resectable esophageal squamous cell carcinoma: a systematic review and meta-analysis based on four randomized controlled trials
Source: Front Oncol. 2025 Aug 13;15:1590111. doi: 10.3389/fonc.2025.1590111 (PMC12381777; doi:10.3389/fonc.2025.1590111)

**Figure 4:** Forest plot comparing neoadjuvant chemotherapy combined with immunotherapy (neoadjuvant chemo-immunotherapy group) and neoadjuvant chemotherapy alone (neoadjuvant chemotherapy group) in esophageal squamous cell carcinoma:(A) Grade 3-4 Adverse Reactions;(B) Neutropenia;(C) Leukopenia;(D)

Thrombocytopenia;(E) Anemia

Grade 3 or 4(**Figure 4 A**)


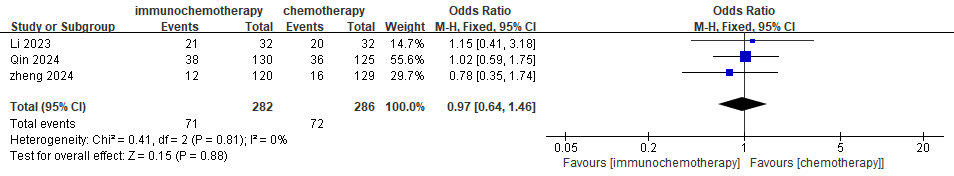


Neutropenia(**Figure 4 B**)


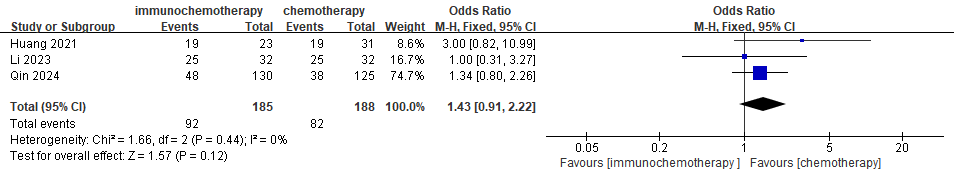


Leukopenia(**Figure 4 C**)


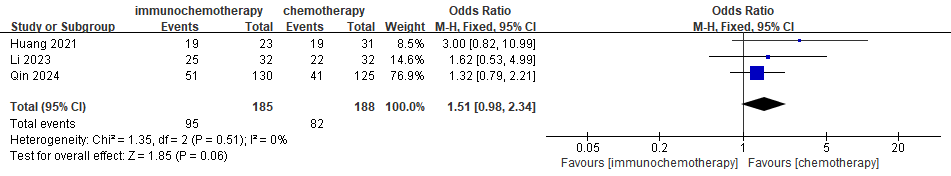


Thrombocytopenia(**Figure 4 D**)


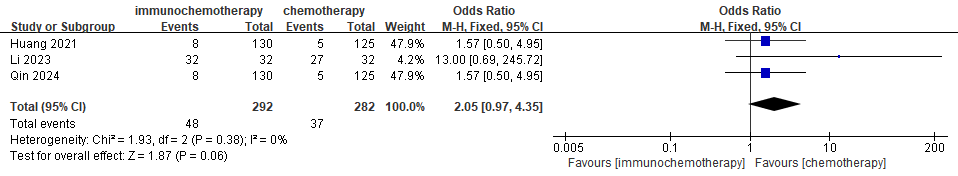


Anemia(**Figure 4 E**)


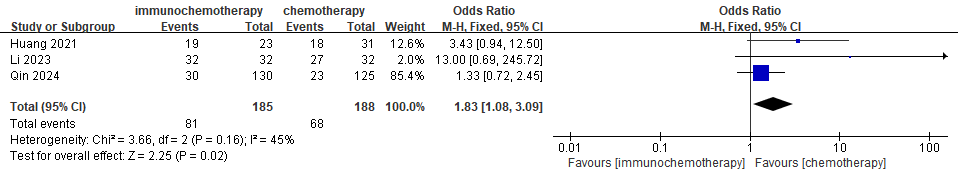


**Figure 5:** Forest plot comparing neoadjuvant chemotherapy combined with immunotherapy (neoadjuvant chemo-immunotherapy group) and neoadjuvant chemotherapy alone (neoadjuvant chemotherapy group) in esophageal squamous cell carcinoma:(A) Nausea;(B) Vomiting;(C) Fatigue;(D) Hair Loss

Nausea(**Figure 5A**)


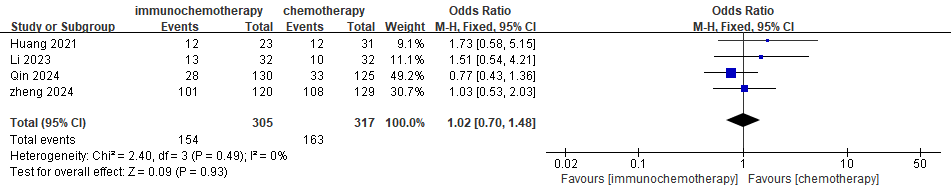


Vomiting(**Figure 5B**)


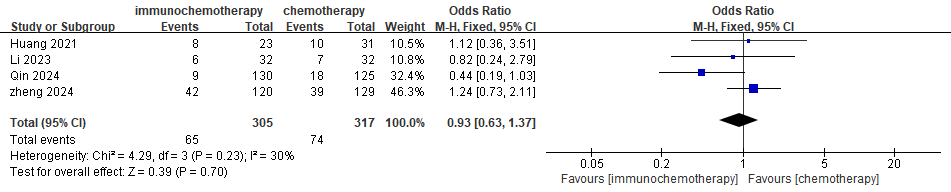


Fatigue(**Figure 5C**)


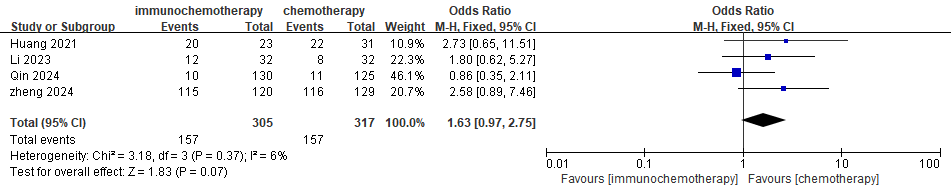


Hair Loss(**Figure 5D**)


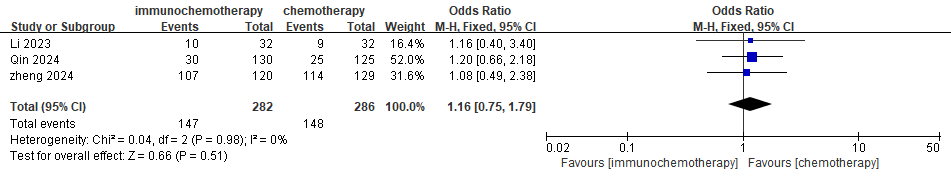


**Figure 6:** Forest plot comparing neoadjuvant chemotherapy combined with immunotherapy (neoadjuvant chemo-immunotherapy group) and neoadjuvant chemotherapy alone (neoadjuvant chemotherapy group) in esophageal squamous cell carcinoma:**(**A) Time from last neoadjuvant dose to definitive surgery (weeks）;

（B）Duration of surgery ；(C)Number of resected lymph nodes; (D)Postoperative complication; (E)Pneumonia；（F）Anastomotic fistula

Time from last neoadjuvant dose to definitive surgery (weeks）(**Figure 6 A)**


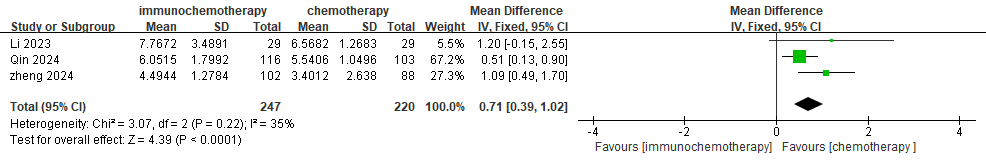


Duration of surgery(**Figure 6 B)**


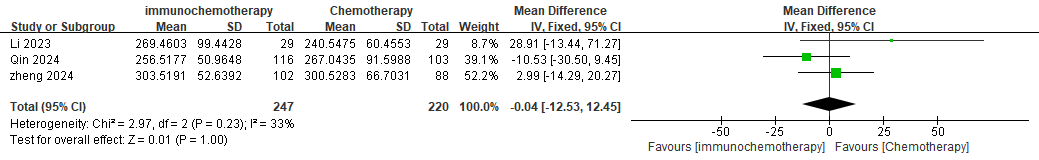


Number of resected lymph nodes(**Figure 6 C)**


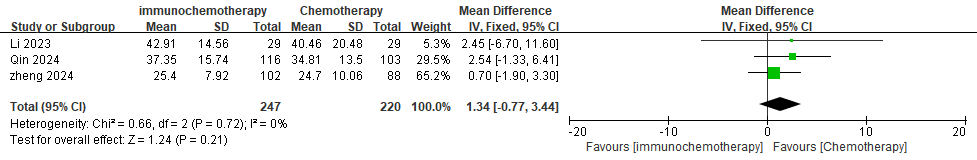


Postoperative complication(**Figure 6 D)**


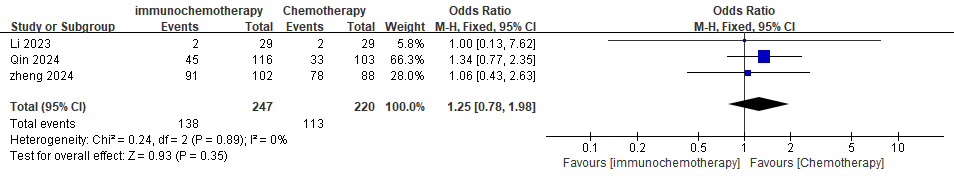


Pneumonia (**Figure 6 E)**


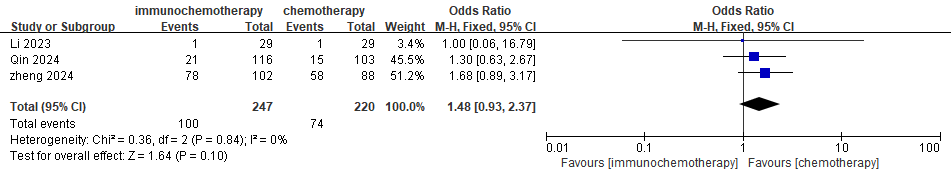


Anastomotic fistula(**Figure 6 F)**


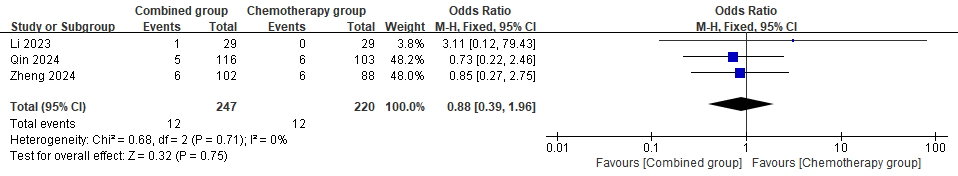


**Figure 7:** Forest plot comparing neoadjuvant chemotherapy combined with immunotherapy (neoadjuvant chemo-immunotherapy group) and neoadjuvant chemotherapy alone (neoadjuvant chemotherapy group) in esophageal squamous cell carcinoma**:**(A) R0 Resection Rate;(B) PCR (Pathological Complete Response);(C) MPR (Major Pathological Response)

R0 Resection Rate (**Figure 7A**)


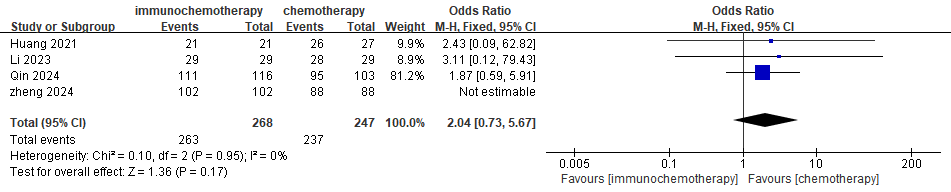


PCR (Pathological Complete Response (**Figure 7B**)


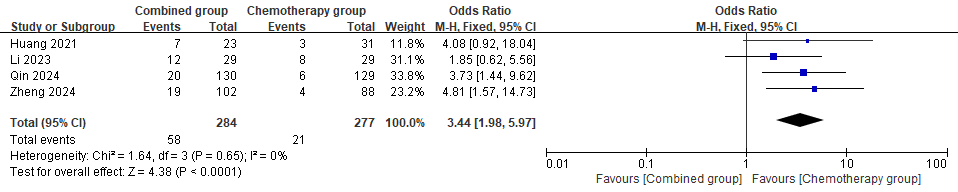


MPR (Major Pathological Response) (**Figure 7C**)


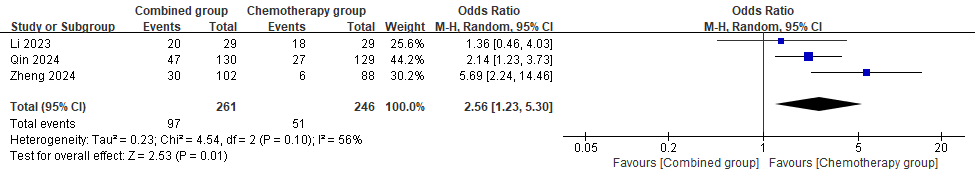


**Figure 2** risk of bias graph: review the authors' judgments on each risk of bias item, expressed as a percentage in all included studies


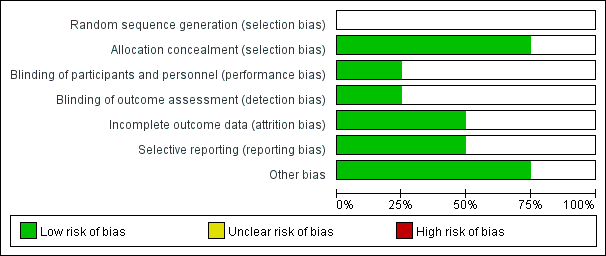


**Figure 3** Risk of Bias Summary:The authors' judgment on each methodological quality item for each included study is reviewed. The symbols "+" (low risk of bias), "-" (high risk of bias), and "blank" (uncertain risk of bias) are used to represent these judgments.
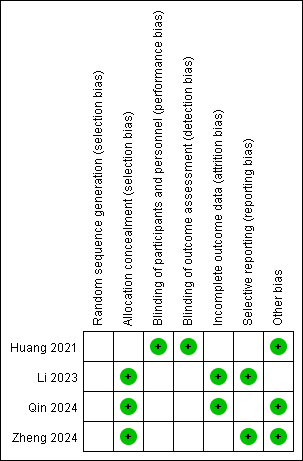

Supplement: Supplementary file 1 [file DataSheet1.docx]
